# A Nomogram for Predicting Lymphovascular Invasion in Superficial Esophageal Squamous Cell Carcinoma

**DOI:** 10.3389/fonc.2021.663802

**Published:** 2021-05-10

**Authors:** Rongwei Ruan, Shengsen Chen, Yali Tao, Jiangping Yu, Danping Zhou, Zhao Cui, Qiwen Shen, Shi Wang

**Affiliations:** Department of Endoscopy, Cancer Hospital of the University of Chinese Academy of Sciences (Zhejiang Cancer Hospital), Institute of Cancer and Basic Medicine (IBMC), Chinese Academy of Sciences, Hangzhou, China

**Keywords:** superficial esophageal squamous cell carcinoma, lymphovascular invasion, risk factor, nomogram, prediction model

## Abstract

The lymphovascular invasion (LVI) status facilitates the determination of the optimal therapeutic strategy for superficial esophageal squamous cell carcinoma (SESCC), but in clinical practice, LVI must be confirmed by postoperative pathology. However, studies of the risk factors for LVI in SESCC are limited. Consequently, this study aimed to identify the risk factors for LVI and use these factors to establish a prediction model. The data of 516 patients who underwent radical esophagectomy between January 2007 and September 2019 were retrospectively collected (training set, n=361, January 2007 to May 2015; validation set, n=155, June 2015 to September 2019). In the training set, least absolute shrinkage and selection operator (LASSO) regression and multivariate analyses were utilized to identify predictive factors for LVI in patients with SESCC. A nomogram was then developed using these predictors. The area under the curve (AUC), calibration curve, and decision curve were used to evaluate the efficiency, accuracy, and clinical utility of the model. LASSO regression indicated that the tumor size, depth of invasion, tumor differentiation, lymph node metastasis (LNM), sex, circumferential extension, the presence of multiple lesions, and the resection margin were correlated with LVI. However, multivariate analysis revealed that only the tumor size, depth of invasion, tumor differentiation, and LNM were independent risk factors for LVI. Incorporating these four variables, model 1 achieved an AUC of 0.817 in predicting LVI. Adding circumferential extension to model 1 did not appreciably change the AUC and integrated discrimination improvement, but led to a significant increase in the net reclassification improvement (p=0.011). A final nomogram was constructed by incorporating tumor size, depth of invasion, tumor differentiation, LNM, and circumferential extension and showed good discrimination (training set, AUC=0.833; validation set, AUC=0.819) and good calibration in the training and validation sets. Decision curve analysis demonstrated that the nomogram was clinically useful in both sets. Thus, it is possible to predict the status of LVI using this nomogram scoring system, which can aid the selection of an appropriate treatment plan.

## Introduction

Esophageal cancer is the seventh most common malignancy in the world (1). For the histopathological type of esophageal cancer, adenocarcinoma account for the majority in western countries, while esophageal squamous carcinoma is the predominate type in China (2, 3). Intraepithelial (Tis), mucosal (T1a), and submucosal (T1b) esophageal squamous cell carcinoma (ESCC), irrespective of lymph node metastasis (LNM), are considered to be superficial esophageal squamous cell carcinoma (SESCC), lack any subjective symptoms, and are associated with a good outcome (4). It is thought that the most effective treatment for esophageal cancers is radical esophagectomy, even for cancers confined to the mucosa (5, 6). However, esophageal cancer has high recurrence rate after radical surgery because of its aggressiveness. Even for the SESCC, postoperative recurrence still happen after esophagectomy with lymph node dissection, remaining the poor prognosis (7, 8).

Lymphovascular invasion (LVI) means tumor cells are present in blood vessels or lymphatics and is only confirmed by histopathological methods (9), which plays an important role in tumor metastasis. Thus, LVI may have significant prognostic value for patients with cancer (10). Indeed, the negative prognostic significance of LVI has been confirmed in breast cancer (11), lung cancer (12), and gastrointestinal tract cancers including esophageal cancer (13–15). Especially, the unfavorable impact of LVI on the postoperative tumor recurrence was also reported (16). However, studies of the risk factors for LVI in SESCC are limited. Therefore, in this study, we aimed to determine the risk factors for LVI in patients with SESCC and establish a nomogram to predict the LVI status to facilitate the selection of an appropriate therapeutic strategy for individuals with esophageal cancer.

## Methods

### Patient Selection and Data Collection

**Figure 1:**
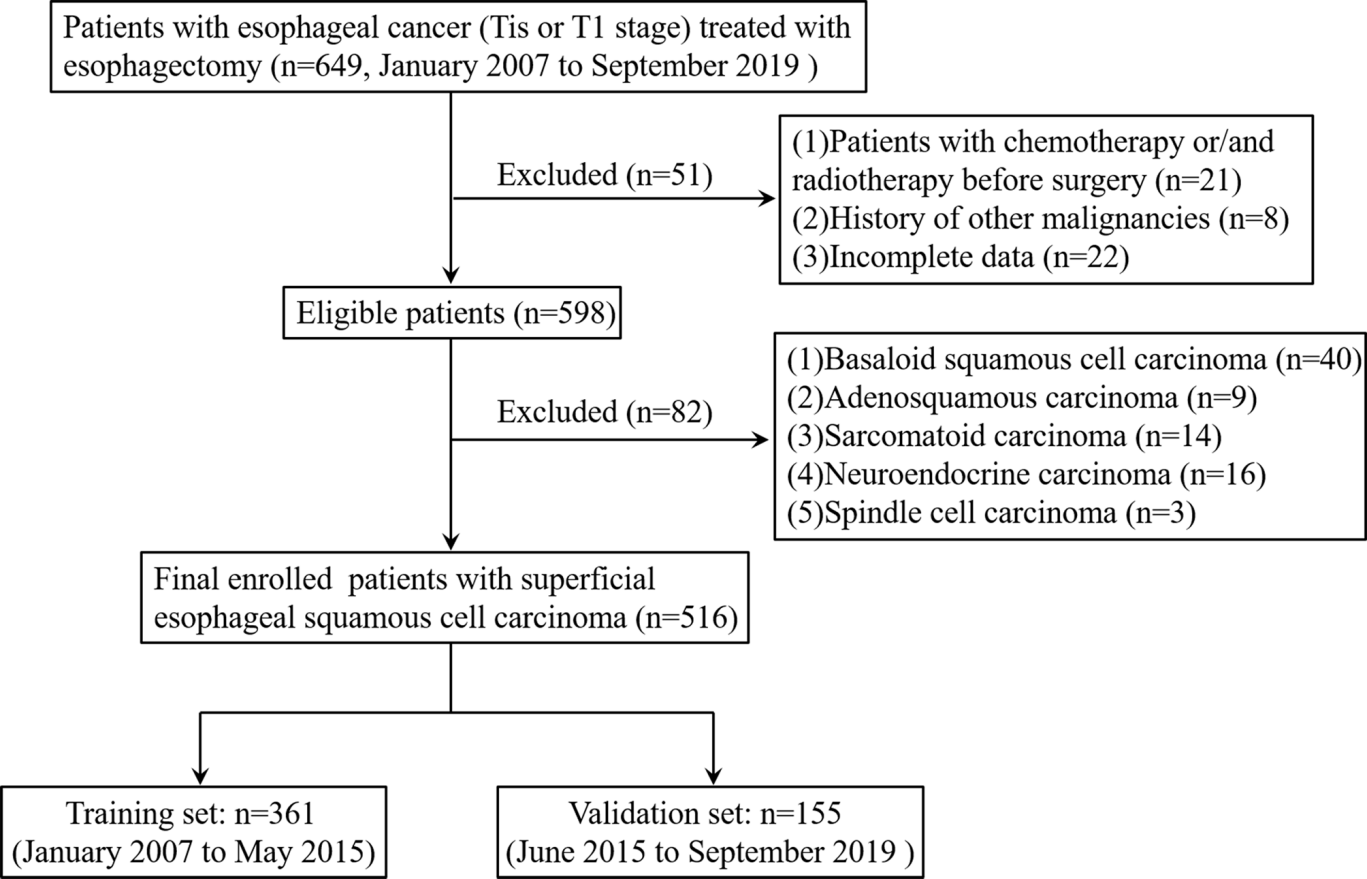
Flowchart of patients included in the analysis.

Between January 2007 and September 2019, the data of patients with histopathologically-confirmed esophageal cancer (Tis or T1 stage) who underwent esophagus resection at Zhejiang Cancer Hospital were retrospectively analyzed. The exclusion criteria were (1) patients who received chemotherapy or radiotherapy before surgery (2); patients with a history of other malignancies or incomplete data; and (3) patients with basaloid squamous cell carcinoma, adenosquamous carcinoma, sarcomatoid carcinoma, neuroendocrine carcinoma, or spindle cell carcinoma. The final eligible patients with SESCC who were admitted between January 2007 and May 2015 were assigned to the training set and those admitted between June 2015 and September 2019 were assigned to the validation set. The flowchart of patient selection is summarized in **Figure 1**.

### Patients Evaluation Before Surgery

Before the operation, the esophagus cancer diagnosis must be confirmed by biopsy and clinical stage need to be evaluated by CT or endoscopic ultrasonography (EUS). Also, the cardio-pulmonary function was evaluated by cardiac ultrasound and pulmonary function test to make sure the patients can tolerate the operation. Finally, some laboratory examinations such as blood routine examination, liver and kidney function, coagulation function, HIV infection, viral hepatitis, and syphilis were evaluated pre-operatively.

### Histopathological Evaluation

Surgical specimens were fixed with formaldehyde and were then cut serially to make slices. The intervals between the tumor tissue and adjacent normal tissues in the slices were 2-5 mm. Tumors that exceed the muscularis mucosa were considered as submucosal invasion (17). We then classified the location of esophageal cancer according to the guidelines of the American Joint Committee on Cancer (18). The portion of the esophagus extending from the entrance of the thoracic cavity to the bifurcation of the trachea is considered the upper esophagus, the section from the trachea bifurcation to the distal esophagus (above the esophagogastric junction) is regarded as the middle esophagus, and the intra-abdominal portion of the esophagus and the junction of the esophagus and stomach constituted the lower esophagus.

### Statistical Analysis

Continuous variables are expressed as the median (range) and were compared using the Mann-Whitney test. Categorical variables were compared using the χ^2^ test or Fisher’s exact test. All variables that were significantly associated with LVI in univariate analysis were candidates for stepwise multivariate logistic analysis. The least absolute shrinkage and selection operator (LASSO) method was used to select the most significant predictive features (19, 20). LASSO regression was performed to identify variables with non-zero coefficients using R with the *glmnet* package. The integrated discrimination improvement (IDI) is the difference in the discrimination slopes for a prediction model with and without one variable, which indicates whether the discrimination slope of a model will improve if one important parameter is added. The net reclassification improvement (NRI) is an index that attempts to quantify how well a new model correctly reclassifies subjects. Typically, this comparison is between an original model and a new model (the original model plus one additional component) (21, 22). The IDI and NRI were calculated using R, version 4.0.3 with the *PredictABEL* package.

According to the results of multivariate logistic regression analysis, we used R software (version 4.0.3) with the *rms* package to formulate a nomogram. The nomogram can proportionally convert each regression coefficient in the logistic regression to a scale of 0 to 100 points (23). The points of each independent variable were summed and the predicted probabilities were derived from the total points. The area under the curve (AUC) and calibration curve were used to assess the predictive performance of this nomogram. In order to evaluate the clinical utility of the nomogram, decision curve analysis (DCA) was performed using R with the *rmda* package. In all analyses, *P*<0.05 was considered to indicate statistical significance. All analyses were performed using SPSS version 22.0 (SPSS Inc, Chicago, Ill) and R, version 4.0.3.

## Results

### Clinicopathological Characteristics

The clinicopathologic characteristics of the 516 included patients are listed in **Table 1** and did not significantly differ between the training (n=361) and validation (n=155) sets. The median age of patients in the training set was 61 (22–78) years and in validation set was 62 (44–79) years. 303 males (83.9%) and 58 females (16.1%) were included into the training set, and 137 males (88.4%) and 18 females (11.6%) were included into validation set. Histopathologically-confirmed LVI was found in 40 patients (11.1%) and 15 patients (9.7%) in the training and validation sets, respectively. The median tumor size was 3 cm in both the training set and the validation set. According to the depth of tumor invasion, 81 patients (22.4%) had mucosal cancer and 280 (77.6%) had submucosal cancer in the training set. In the validation set, 37 patients (23.9%) had mucosal cancer and 118 (76.1%) had submucosal cancer. LNM was found in 87 patients (24.1%) in the training set and 38 patients (24.5%) in the validation set.

**Table 1:** Participant characteristics.

Variables	Training set (n=361)	Validation set (n=155)	P
Sex, n(%)			0.223
Male	303(83.9)	137(88.4)	
Female	58(16.1)	18(11.6)	
Age(years), median(range)	61(22-78)	62(44-79)	0.107
Tumor size(cm), median(range)	3(1-9)	3(1-11)	0.116
Circumferential extension, n(%)			0.343
≤1/2	291(80.6)	119(76.8)	
>1/2	70(19.4)	36(23.2)	
Location within esophagus, n(%)			0.341
Upper	15(4.2)	3(1.9)	
Middle	257(71.2)	108(69.7)	
Lower	89(24.6)	44(28.4)	
Depth of invasion, n(%)			0.732
Mucosa	81(22.4)	37(23.9)	
Submucosa	280(77.6)	118(76.1)	
Tumor differentiation, n(%)			0.215
Carcinoma in situ	13(3.6)	3(1.9)	
Well	68(18.8)	30(19.4)	
Moderate	179(49.6)	66(42.6)	
Poor	101(28.0)	56(36.1)	
LVI, n(%)			0.756
No	321(88.9)	140(90.3)	
Yes	40(11.1)	15(9.7)	
Macroscopic type, n(%)			0.771
I	156(43.2)	64(41.3)	
II	191(52.9)	83(53.5)	
III	14(3.9)	8(5.2)	
Multiple lesions, n(%)			0.996
No	333(92.2)	143(92.3)	
Yes	28(7.8)	12(7.7)	
LNM			0.919
No	274(75.9)	117(75.5)	
Yes	87(24.1)	38(24.5)	
Resection margin, n(%)			0.419
R0	347(96.1)	152(98.1)	
R1	14(3.9)	3(1.9)	

LVI, lymphovascular invasion; LNM, Lymph node metastasis;

I = superficial and protruding type; II = flat type; III = superficial and excavated type;

P: Categorical variables—χ^2^ test or Fisher’s exact test; Continuous variables—Mann-Whitney test.

### Independent Risk Factors for LVI

Comparisons of the clinicopathological characteristics between the LVI-positive and -negative groups are summarized in **Table 2**. Variables such as tumor size, tumor invasion depth, tumor differentiation, and LNM, were significantly associated with the LVI according to the univariate analysis (**Table 2**). However, age, sex, circumferential extension, tumor location, the presence of multiple lesions, and the resection margin were not correlated with LVI. Furthermore, ROC curve analysis indicated that the tumor size cutoff value was 2.5 cm in training set (**Figure S1**). Tumor size, tumor invasion depth, tumor differentiation, and LNM were identified as independent predictive factors of LVI in the multivariate analysis (**Table 3**). The LVI rates according to the risk factors based on the results of multivariate logistic analysis are summarized in **Tables S1** and **S2**. Patients with tumors of >2.5 cm in size, submucosal invasion, LNM, and poor tumor differentiation seemed to have high LVI rate.

**Table 2:** Clinicopathologic findings according to lymphovascular invasion in training set.

Variables	LVI(-) (n=321)	LVI(+) (n=40)	P
Gender, n(%)			0.362
Male	267(83.2)	36(90.0)	
Female	54(16.8)	4(10.0)	
Age(years), median(range)	61(22-78)	58(48-76)	0.260
Tumor size(cm), median(range)	3(1-9)	4(2-7)	**0.042**
Circumferential extension, n(%)			0.456
≤1/2	257(80.1)	34(85.0)	
>1/2	64(19.9)	6(15.0)	
Location within esophagus, n(%)			0.797
Upper	14(4.4)	1(2.5)	
Middle	229(71.3)	28(70.0)	
Lower	78(24.3)	11(27.5)	
Depth of invasion, n(%)			**0.005**
Mucosa	79(24.6)	2(5.0)	
Submucosa	242(75.4)	38(95.0)	
Tumor differentiation, n(%)			**0.007**
Carcinoma in situ	13(4.0)	0(0)	
Well	64(19.9)	4(10.0)	
Moderate	163(50.8)	16(40.0)	
Poor	81(25.2)	20(50.0)	
LNM, n(%)			**<0.001**
No	258(80.4)	16(40.0)	
Yes	63(19.6)	24(60.0)	
Macroscopic type, n(%)			0.222
I	134(41.7)	22(55.0)	
II	175(54.5)	16(40.0)	
III	12(3.7)	2(5.0)	
Multiple lesions, n(%)			0.069
No	299(93.1)	34(85.0)	
Yes	22(6.9)	6(15.0)	
Resection margin, n(%)			0.632
R0	308	39	
R1	13	1	

LVI, lymphovascular invasion; LNM, Lymph node metastasis;

I = superficial and protruding type; II = flat type; III = superficial and excavated type;

P: Categorical variables—χ^2^ test or Fisher’s exact test; Continuous variables—Mann-Whitney test. The bold values mean statistical significance.

**Table 3:** Multivariate logistic analysis of risk factors for lymphovascular invasion in training set.

Factors	β	OR	95% CI	P
Tumor size				
≤2.5cm	Reference			
>2.5cm	1.159	3.186	1.348-7.532	**0.008**
Depth of invasion				
Mucosa	Reference			
Submucosa	1.583	4.871	1.082-21.925	**0.039**
Tumor differentiation				
Well or Carcinoma in situ	-1.619	0.198	0.059-0.659	**0.008**
Moderate	-0.906	0.404	0.182-0.896	**0.026**
Poor	Reference			
LNM				
No	Reference			
Yes	1.422	4.145	1.991-8.629	**<0.001**
Multiple lesions, n (%)				
No	Reference			
Yes	0.231	1.260	0.411-3.859	0.686

LNM, Lymph node metastasis; I = superficial and protruding type; II = flat type; III = superficial and excavated type. The bold values mean statistical significance.

### Identification of Predictive Factors by LASSO Regression

**Figure 2:**
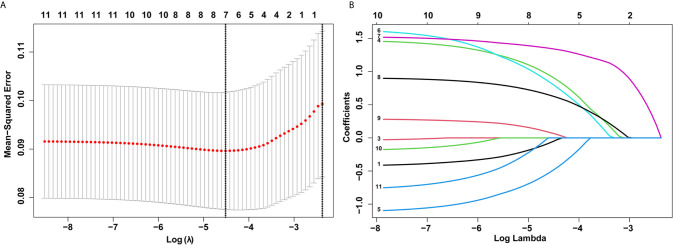
Selection of demographic and clinical features using the least absolute shrinkage and selection operator (LASSO) regression model. **(A)** Selection of optimal parameters (lambda) from the LASSO model using 10-fold cross-validation and minimum criteria. Dotted vertical lines were drawn at the optimal values using the minimum criteria and the 1 standard error of the minimum criteria (1-SE criteria). **(B)** LASSO coefficient profiles of 10 features. A coefficient profile plot was produced against the log (lambda) sequence.

In total, 11 characteristics were analyzed by LASSO regression in the training set, and seven candidate variables were determined to be associated with LVI (**Figure 2**). The weights for each factor associated with LVI were obtained by calculating the coefficients when log (λ) = -1.961 and λ = 0.0109 in the LASSO regression model (**Figures 2A**, **B**). The coefficients for each parameter were as follows: -0.0546 for sex, 0.9130 for tumor size, 0.4897 for circumferential extension, 0.8064 for depth of invasion, 1.3439 for LNM, 0.6298 for tumor differentiation, and 0.0693 for multiple lesions.

### Confirmation of the Best Prediction Model for LVI

The base model (model 1) was then created by incorporating the four variables determined to be associated with LVI (tumor size, tumor invasion depth, tumor differentiation, and LNM). By adding sex, circumferential extension, and multiple lesions in sequence to the model 1, we constructed three new models named model 2, model 3, and model 4 (**Table 4**). Using model 1 as the reference, model 2 and model 4 did not exhibit superiority for predicting LVI. Interestingly, adding circumferential extension to model 1 did not appreciably change the AUC and IDI, but led to a significant improvement in the NRI (**Table 4**), indicating that model 3 was superior to model 1 and that circumferential extension can also be considered a predictive factor of LVI. The reclassification of patients with and without LVI are provided in **Table S3**.

**Table 4:** Comparison of different prediction model for estimating the risk of LVI presence.

Variables	AUC (95%CI)	P	IDI% (95%CI)	P	NRI% (95%CI)	P
Model 1(Base model)	0.817(0.757-0.878)	Reference		Reference		Reference
Model 2	0.819(0.757-0.880)	0.869	0.36(-0.40-1.13)	0.351	4.70(-4.91-14.32)	0.338
Model 3	0.833(0.776-0.891)	0.134	1.55(-0.68-3.77)	0.173	17.20(4.03-30.37)	**0.011**
Model 4	0.817(0.756-0.878)	0.931	0.12(-0.38-0.62)	0.641	-0.31(-0.92-0.30)	0.317

AUC, area under curve; IDI, integrated discrimination improvement; NRI, net re-classification improvement.

Model 1=Tumor size+ Depth of invasion+ Tumor differentiation+ LNM;

Model 2=Model 1+ Gender;

Model 3=Model 1+ Circumferential extension;

Model 4=Model 1+ Multiple lesions.

The bold value means statistical significance.

### Development and Validation of an LVI-Predicting Nomogram

**Figure 3:**
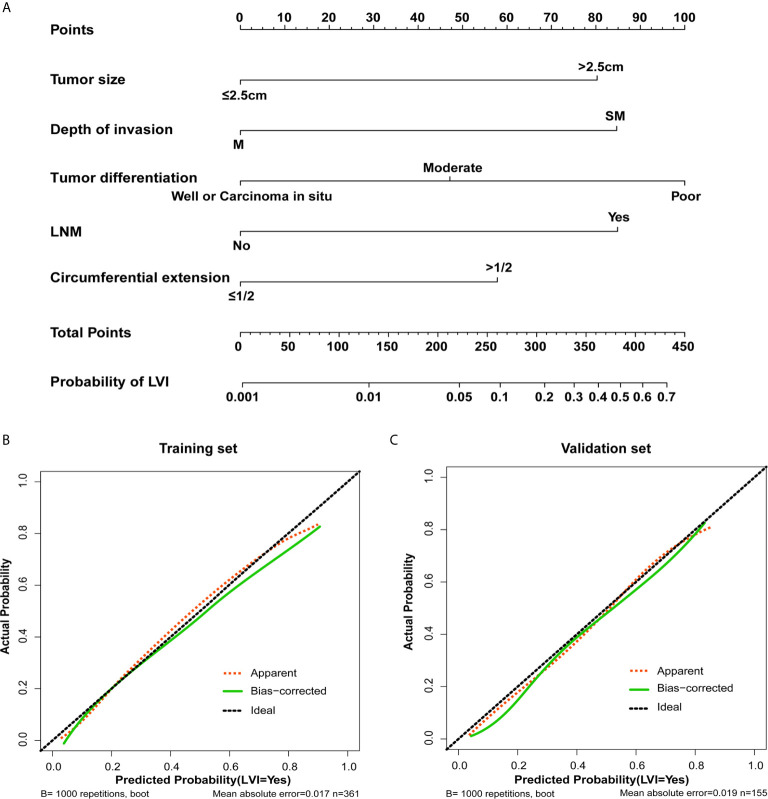
The nomogram and its calibration. **(A)** Nomogram for predicting the probability of lymphovascular invasion in patients with superficial esophageal squamous cell carcinoma in training set. Locate the patient’s characteristic on a variable row and draw a vertical line straight up to the points’ row (top) to assign a point value for the variable. Add up the total number of points and drop a vertical line from the total points’ row to obtain the probability of lymph node metastasis. The calibration curve based on internal validation with a bootstrap resampling frequency of 1000 in the training cohort **(B)** and validation set **(C)**.

**Figure 4:**
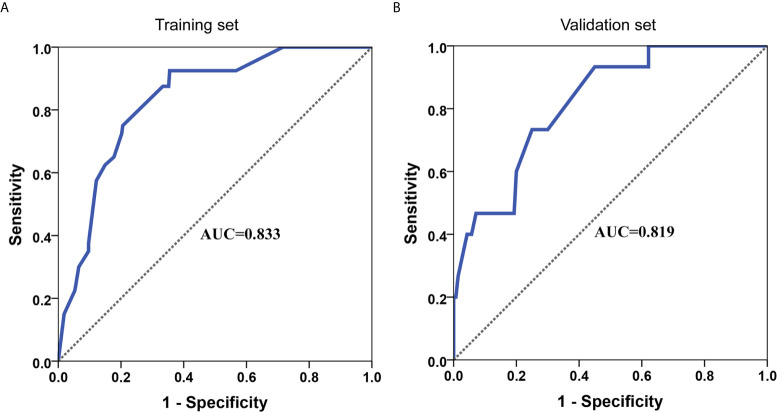
ROC curve of the nomogram for predicting LVI in training set **(A)** and validation set **(B)**.

A nomogram for LVI prediction was formed by incorporating five variables—— tumor size, tumor invasion depth, tumor differentiation, LNM, and circumferential extension (**Figure 3A**). The nomogram was validated by internal (bootstrap method) and external validation (validation set). This nomogram showed a good performance for predicting LVI risk, with an AUC of 0.833 (**Figure 4A**). Additionally, a calibration curve of the training set demonstrated good consistency between the predicted and observed results regarding the LVI status (**Figure 3B**). In the validation set, the nomogram achieved an AUC of 0.819 for the estimation of LVI risk (**Figure 4B**), and its calibration curve also fitted well (**Figure 3C**).

### The Nomogram Score System for LVI Risk Prediction and Clinical Use

**Figure 5:**
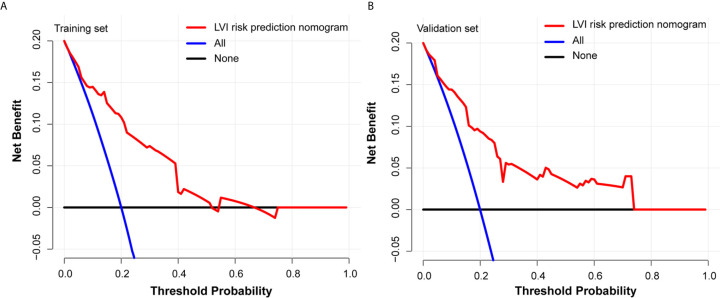
Decision curves of the nomogram predicting LVI in training set **(A)** and validation set **(B)**.

Each predictive variable displayed in the nomogram was assigned a risk score. The detailed scores of the five variables (tumor size, tumor invasion depth, tumor differentiation, LNM, and circumferential extension) in the training sets are presented in **Figure 3A** and **Table S4**. We predicted the presence of LVI by summing the scores of these five variables, and the final total scores ranged from 0 to 408 in the training set. The optimal cutoff point of the total nomogram score for LVI in the training set was determined to be 243 according to the ROC curve analysis (**Table S5**). As a result, patients with total scores of ≤243 in the training set were classified as low risk and patients with total scores of >243 were classified as high risk. In addition, the DCA in the training and validation sets indicated that our nomogram had significant net benefits for almost all threshold probabilities at different points, suggesting a good clinical utility of this nomogram (**Figure 5**).

## Discussion

SESCC just invade the mucosa and submucosa and lack of any subjective symptoms. Hence, early diagnosis is difficult for these patients, and most esophageal cancers are at a locally advanced stage when the diagnosis is confirmed. However, due to advancements in diagnostic techniques and the widespread use of endoscopic screening, the rate of SESCC diagnosis is increasing (24). In this study, we aimed to identify predictors of LVI in order to aid determination of the optimal treatment strategy for SESCC. Our findings indicated that patients with LVI were significantly more likely to have larger tumors, poorer differentiation, deeper tumor invasion, and LNM. Circumferential extension was also determined to be associated with LVI in the LASSO regression analysis, but lost significance in the multivariate analysis of the training set.

We measured the largest tumor diameter under a microscope as tumor size, then used univariate and multivariate analyses to investigate the association between tumor size and LVI. It can be concluded from our study that tumor size was significantly correlated with LVI and was also identified as an important predictor of LVI. Although SESCC comprises both mucosal and submucosal cancers, the LVI status may differ between mucosal and submucosal infiltration. Taking mucosal infiltration as reference, the odds ratio of the submucosal infiltration was 4.871 for prediction of LVI in our training set (**Table 3**), demonstrating that the presence of submucosal infiltration was identified as a significant risk factor of LVI. The LVI rate among SESCC patients with mucosal cancer was 2.47% (2/81), while the incidence of LVI increased dramatically to 13.57% (38/280) in patients with submucosal invasion (**Table S1**).

It was previously reported that histological differentiation was related to LVI (25). Consistently, we also found a significant association between tumor differentiation and LVI in the current study (**Table 3**). LVI is an essential and important step in the development of LNM and systemic dissemination of cancer cells (26). Thus, LVI is closely related to LNM. In clinical practice, LVI is usually postoperatively diagnosed by histopathology, but LNM can be detected by imaging techniques such as computed tomography (CT), magnetic resonance imaging (MRI), positron emission tomography (PET)/CT, and ultrasound (27–29). PET/CT in particular offers high accuracy in the diagnosis of LNM (30–32). Therefore, we wished to use LNM to predict LVI, and finally found that LNM was significantly related to LVI in patients with SESCC (**Table 3**).

Interestingly, according to the LASSO regression analysis, seven variables (sex, tumor size, circumferential extension, invasion depth, LNM, tumor differentiation, and multiple lesions) were associated with LVI when log (λ) = -1.961 and λ = 0.0109 (**Figures 2A, B**). However, sex, circumferential extension, and multiple lesions were not related to LVI in the univariate and multivariate analyses. In order to explore the relationships between these three variables and LVI, we further constructed three new models named model 2, model 3, and model 4 by adding sex, circumferential extension, and multiple lesions, respectively, to the base model. Compared with model 1, model 2 and model 4 did not exhibit any superiority for LVI prediction. Furthermore, the addition of circumferential extension to the base model did not improve the AUC or IDI for predicting LVI, but the NRI values significantly improved (**Table 4**), indicating that circumferential extension could be considered as a risk factor for LVI.

Moreover, a nomogram was developed for LVI prediction by incorporating the five independent predictors (tumor size, tumor invasion depth, tumor differentiation, LNM, and circumferential extension), with an AUC of 0.833 in training set and 0.819 in the validation set **(** **Figure 4**). The calibration curves also showed good consistency between the predicted results and actual observation results, implying that the nomogram had high accuracy for predicting LVI **(** **Figures 3B, C**). Thereafter, the sensitivity and specificity of this nomogram for estimating the LVI risk were summarized, and the cutoff value of 243 points was identified in the training set (**Table S5**) according to the maximum Youden index (derived from the sensitivity and specificity). Patients with a total score of >243 in the training set were considered high-risk and patients with a total score of ≤243 were considered low-risk.

The most important and final line of evidence for the use of the nomogram is based on the need to interpret individual requirements with regard to additional treatment or care. Therefore, to confirm the clinical utility, we determined the decisions assisted by this nomogram whether improved patients’ consequences by using DCA. The clinical outcomes based on the threshold probability can be known from this new method. False positive proportion was subtracted from the true positives proportion, and then the relative risk of false positive and false negative results was weighted to obtain the net benefit (33). It can be gotten from the decision curve that if the threshold probability of a patient was >20%, more benefit was added than either the scheme of treating all patients or the scheme of treating zero patient by using our nomogram to predict LVI.

In summary, tumor size, tumor invasion depth, tumor differentiation, and LNM were identified as significant predictive factors for LVI in patients with SESCC. Circumferential extension was also identified as a predictor for LVI by calculating the IDI and NRI. Furthermore, a nomogram scoring system was established using these five variables, making individualized LVI prediction easier and facilitating optimal treatment strategy selection for patients with SESCC. However, there are some limitations in this study. First, this was a retrospective study based on data from a single institution. This design could pose some potential biases including selection. Second, the sample size was relatively small, which restricted some of the analyses. Therefore, it is necessary to validate the results using data from multiple centers and a prospective study with big sample size is required to further confirm the reliability of the nomogram. Last but not least, our nomogram may improve and facilitate treatment strategy selection, which may lead to early diagnosis and prompt treatment initiation for patients with SESCC.

## Data Availability Statement

The raw data supporting the conclusions of this article will be made available by the authors, without undue reservation.

## Ethics Statement

The studies involving human participants were reviewed and approved by Ethics committee of Zhejiang Cancer Hospital. The patients/participants provided their written informed consent to participate in this study.

## Author Contributions

SW and RR conceived the idea and designed study. YT, JY, DZ, and ZC collected data. RR and QS analyzed the data. RR and SC drafted the manuscript. All authors contributed to the article and approved the submitted version.

## Conflict of Interest

The authors declare that the research was conducted in the absence of any commercial or financial relationships that could be construed as a potential conflict of interest.
