# Supplementary material for: A Nomogram for Predicting Lymphovascular Invasion in Superficial Esophageal Squamous Cell Carcinoma
Source: Front Oncol. 2021 May 10;11:663802. doi: 10.3389/fonc.2021.663802 (PMC8141657; doi:10.3389/fonc.2021.663802)
Supplement: Supplementary file 1 [file DataSheet_1.docx]

**A nomogram for predicting lymphovascular invasion in superficial esophageal squamous cell carcinoma**

Rongwei Ruan^†^,Shengsen Chen^†^,Yali Tao^†^,Jiangping Yu, Danping Zhou, Zhao Cui, Qiwen Shen, Shi Wang*

Department of Endoscopy, Cancer Hospital of the University of Chinese Academy of Sciences(Zhejiang Cancer Hospital), Institute of Cancer and Basic Medicine(IBMC), Chinese Academy of Sciences, Hangzhou 310022, Zhejiang, China.

^†^Rongwei Ruan, Shengsen Chen and Yali Tao contributed equally to this work.

*Corresponding author: Shi Wang. Department of Endoscopy, Cancer Hospital of the University of Chinese Academy of Sciences(Zhejiang Cancer Hospital), Institute of Cancer and Basic Medicine(IBMC), Chinese Academy of Sciences, Hangzhou 310022, Zhejiang, China.

E-mail: wangshi@zjcc.org.cn, telephone number: +86-571-88122277, fax number: +86-571-88122277.

**Figure legends**

**Figure S1.** Determination of the optimal cutoff value for tumor size based on the ROC analysis in training set.


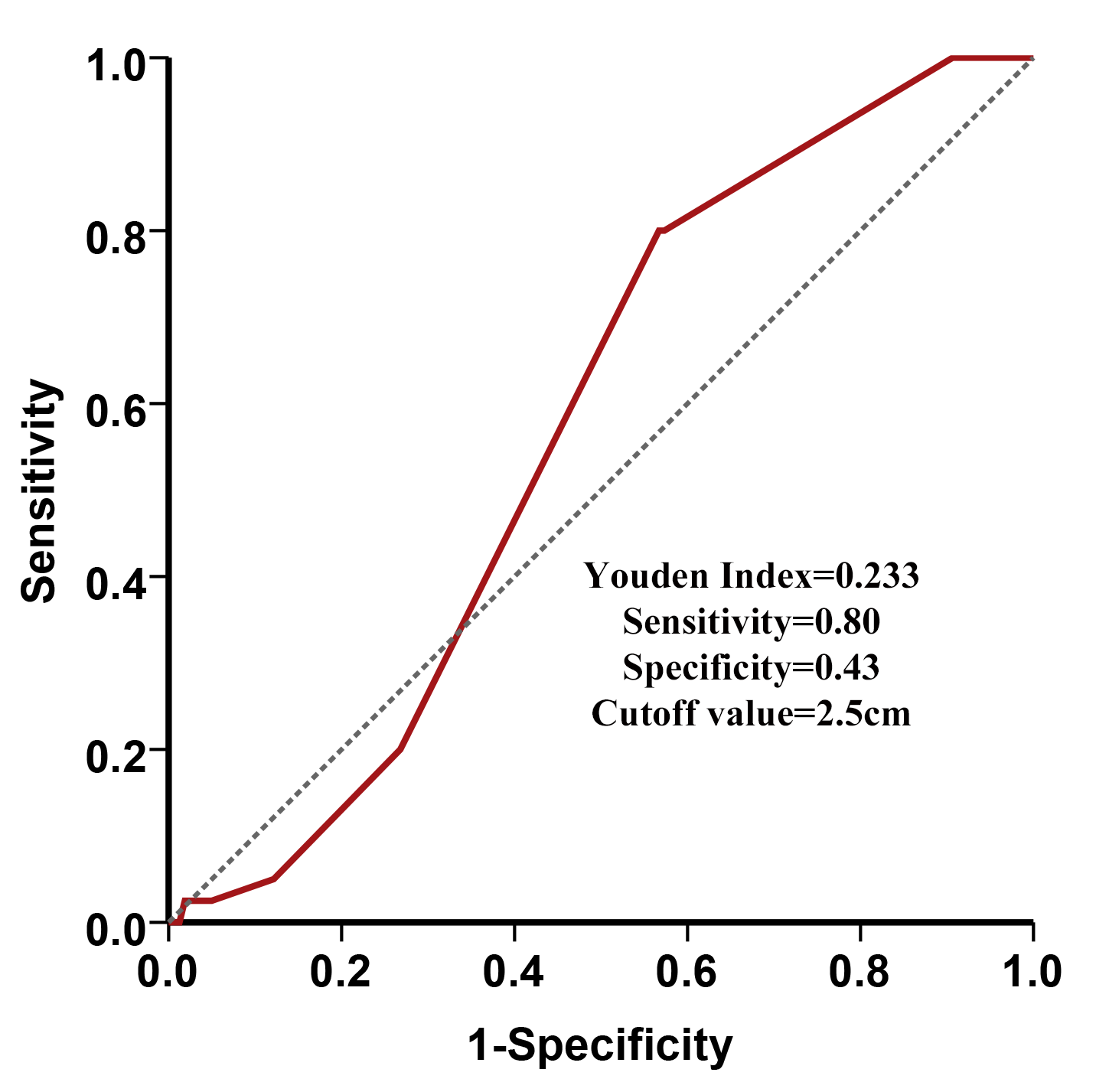


**Figure S1**

| TableS2. Lymphovascular invasion rates according to tumor size, depth of invasion, and lymph node metastasis | | |
| --- | --- | --- |
|  | LNM (-) LVI, n (%) | LNM (+) LVI, n (%) |
| Tumor size |  |  |
| Mucosal invasion(n=81) |  |  |
| ≤2.5cm | 0/28(0) | 0/3(0) |
| >2.5cm | 0/47(0) | 2/3(66.7) |
| Submucosal invasion(n=280) |  |  |
| ≤2.5cm | 3/92(3.3) | 5/24(20.8) |
| >2.5cm | 13/107(12.1) | 17/57(29.8) |

| TableS1. Lymphovascular invasion rates according to tumor differentiation and depth of invasion | | | | |
| --- | --- | --- | --- | --- |
| Tumor differentiation | M1 LVI, n (%) | M2 LVI, n (%) | M3 LVI, n (%) | SM LVI, n (%) |
| Carcinoma in situ | 0/13(0) | 0/0 | 0/0 | 0/0 |
| Well | 0/0 | 0/0 | 0/10(0) | 4/58(6.9) |
| Moderate | 0/0 | 0/0 | 1/30(3.3) | 15/149(10.1) |
| Poor | 0/0 | 0/0 | 1/28(3.6) | 19/73(26.0) |

| Table S3. Predicted risk of LVI events using a multivariate risk prediction model with and without inclusion of circumferential extension data in training set | | | | |
| --- | --- | --- | --- | --- |
| Model 1 (without circumferential extension) | Model 3 (with circumferential extension) | | | |
| Frequency, % | <30 | 30-60 | >60 | Total |
|  | Participants who experience a LVI event, n | | | |
| <30 | 24 | 7 | 0 | 31 |
| 30-60 | 0 | 7 | 2 | 9 |
| >60 | 0 | 0 | 0 | 0 |
| Total | 24 | 14 | 2 | 40 |
|  | Participants who do not experience a LVI event, n | | | |
| <30 | 291 | 20 | 0 | 311 |
| 30-60 | 2 | 8 | 0 | 10 |
| >60 | 0 | 0 | 0 | 0 |
| Total | 293 | 28 | 0 | 321 |

| TableS4. Risk scores based on nomogram for LVI prediction in training set | | | | | | |
| --- | --- | --- | --- | --- | --- | --- |
| Factor | Risk scores | | | | | |
|  | 0 | 47 | 58 | 80 | 85 | 100 |
| Tumor size(cm) | ≤2.5cm |  |  | >2.5cm |  |  |
| Depth of invasion | Mucosa |  |  |  | Submucosa |  |
| Tumor differentiation | Well or Carcinoma in situ | Moderate |  |  |  | Poor |
| LNM | No |  |  |  | Yes |  |
| Circumferential extension | ≤1/2 |  | >1/2 |  |  |  |

| TableS5. Identification of the optimal cutoff value of the total nomogram scores in the ROC curve in training set | | | | | |
| --- | --- | --- | --- | --- | --- |
| Criterion | Sensitivity% | 95% CI | Specificity% | 95% CI | Youden Index |
| ≥0 | 100.00 | 91.2 - 100.0 | 0.00 | 0.0 - 1.1 | 0 |
| >0 | 100.00 | 91.2 - 100.0 | 0.31 | 0.008 - 1.7 | 0.0031 |
| >47 | 100.00 | 91.2 - 100.0 | 0.62 | 0.08 - 2.2 | 0.0062 |
| >58 | 100.00 | 91.2 - 100.0 | 2.80 | 1.3 - 5.3 | 0.028 |
| >80 | 100.00 | 91.2 - 100.0 | 4.36 | 2.4 - 7.2 | 0.0436 |
| >105 | 100.00 | 91.2 - 100.0 | 7.48 | 4.8 - 10.9 | 0.0748 |
| >127 | 100.00 | 91.2 - 100.0 | 10.28 | 7.2 - 14.1 | 0.1028 |
| >132 | 100.00 | 91.2 - 100.0 | 10.90 | 7.7 - 14.8 | 0.109 |
| >138 | 100.00 | 91.2 - 100.0 | 13.71 | 10.1 - 18.0 | 0.1371 |
| >143 | 100.00 | 91.2 - 100.0 | 19.94 | 15.7 - 24.7 | 0.1994 |
| >158 | 100.00 | 91.2 - 100.0 | 22.74 | 18.3 - 27.7 | 0.2274 |
| >165 | 100.00 | 91.2 - 100.0 | 23.68 | 19.1 - 28.7 | 0.2368 |
| >180 | 100.00 | 91.2 - 100.0 | 26.17 | 21.4 - 31.3 | 0.2617 |
| >185 | 100.00 | 91.2 - 100.0 | 28.35 | 23.5 - 33.6 | 0.2835 |
| >190 | 92.50 | 79.6 - 98.4 | 43.61 | 38.1 - 49.2 | 0.3611 |
| >212 | 92.50 | 79.6 - 98.4 | 48.29 | 42.7 - 53.9 | 0.4079 |
| >223 | 92.50 | 79.6 - 98.4 | 54.83 | 49.2 - 60.4 | 0.4733 |
| >228 | 92.50 | 79.6 - 98.4 | 55.14 | 49.5 - 60.7 | 0.4764 |
| >238 | 92.50 | 79.6 - 98.4 | 57.94 | 52.3 - 63.4 | 0.5044 |
| **>****243** | **92.50** | **79.6 - 98.4** | **64.49** | **59.0 - 69.7** | **0.5699** |
| >250 | 87.50 | 73.2 - 95.8 | 64.80 | 59.3 - 70.0 | 0.523 |
| >265 | 87.50 | 73.2 - 95.8 | 66.67 | 61.2 - 71.8 | 0.5417 |
| >270 | 72.50 | 56.1 - 85.4 | 79.75 | 74.9 - 84.0 | 0.5225 |
| >275 | 65.00 | 48.3 - 79.4 | 82.24 | 77.6 - 86.3 | 0.4724 |
| >297 | 62.50 | 45.8 - 77.3 | 85.05 | 80.7 - 88.8 | 0.4755 |
| >308 | 57.50 | 40.9 - 73.0 | 87.85 | 83.8 - 91.2 | 0.4535 |
| >323 | 35.00 | 20.6 - 51.7 | 90.34 | 86.6 - 93.3 | 0.2534 |
| >328 | 30.00 | 16.6 - 46.5 | 93.46 | 90.2 - 95.9 | 0.2346 |
| >350 | 22.50 | 10.8 - 38.5 | 94.70 | 91.7 - 96.9 | 0.172 |
| >355 | 15.00 | 5.7 - 29.8 | 98.13 | 96.0 - 99.3 | 0.1313 |
| >408 | 0.00 | 0.0 - 8.8 | 100.00 | 98.9 - 100.0 | 0 |
